# A review on the pharmacology of cariprazine and its role in the treatment of negative symptoms of schizophrenia

**DOI:** 10.3389/fpsyt.2024.1385925

**Published:** 2024-04-22

**Authors:** Panneer Selvan, Prashant Devkare, Arthik Shetty, Shruti Dharmadhikari, Chintan Khandhedia, Amey Mane, Suyog Mehta, Chittaranjan Andrade

**Affiliations:** ^1^ Department of Psychiatry, Sneka Mind Care Hospital, Tirunelveli, Tamil Nadu, India; ^2^ Medical Affairs and Clinical Research, Sun Pharmaceutical Industries Limited, Mumbai, India; ^3^ Medical Affairs and Clinical Research, Sun Pharma Laboratories Limited, Mumbai, India; ^4^ Department of Clinical Psychopharmacology and Neurotoxicology, National Institute of Mental Health and Neurosciences (NIMHANS), Bangalore, India

**Keywords:** atypical antipsychotics, cariprazine, negative symptoms, schizophrenia, pharmacology, socializing drug, third-generation antipsychotics, D3 receptor

## Abstract

Management of negative symptoms is one of the most challenging and important unmet needs of schizophrenia treatment. Negative symptoms together with positive symptoms result in significant psychosocial impairment and poor quality of life. Existing studies on atypical antipsychotics reported limited treatment adherence due to higher prevalence of treatment-emergent adverse events, such as diabetes, weight gain, hyperlipidemia, hyperprolactinemia and hypertension. A compound with greater affinity for dopamine D2/D3 receptors may improve negative symptoms, mood, and cognitive impairment associated with schizophrenia. In 2015, the US FDA has approved cariprazine, a partial D2/D3 agonist for treatment of schizophrenia, mania or mixed episodes. Midlands and Lancashire Commissioning Support Unit, UK (2019) has particularly suggested cariprazine for the treatment of predominant negative symptoms of schizophrenia. India’s Central Drugs Standard Control Organization (CDSCO) has approved cariprazine in 2021 for the treatment of schizophrenia, manic or mixed episodes associated with bipolar I disorder. A ten-fold greater affinity for D3 receptors and partial agonism to serotonin receptors, along with longer half-life make cariprazine distinct when compared with other atypical antipsychotics. Cariprazine is also reported to have fewer incidents of metabolic and hormonal adverse events, and has been shown to provide better relapse prevention. Recent evidence indicates promising effect of cariprazine in ameliorating negative symptoms as well as psychotic symptoms in patients with schizophrenia. In addition, improved adherence to treatment (adjunctive/monotherapy) with cariprazine in patients having inadequate response to an ongoing antipsychotic treatment has also been clinically established. This review presents the evidence-based safety and efficacy of cariprazine for treatment of predominant negative symptoms of schizophrenia.

## 1 Introduction

Schizophrenia is a complex neuropsychiatric disorder, presenting with positive (hallucinations and delusions), negative (blunted affect, alogia, anhedonia, asociality and avolition), and cognitive (impaired retrieval of information like thinking, learning and memorizing) symptoms (1). In addition to these symptoms, patients with schizophrenia often experience affective symptoms (depression and anxiety) that is associated with increased risk of suicide and poor quality of life (2). Positive symptoms are primarily monitored to diagnose an active state of schizophrenia, but negative symptoms are significant contributors of poor psychosocial functioning and performance, impacting the patient’s quality of life (3). Identification of negative symptoms can be sometimes challenging due to their insidious onset, paucity of psychotic signs and similarity with other clinical features of schizophrenia, resulting in delayed treatment outcomes (1). The fundamental pathophysiological mechanism of negative symptoms is different from positive symptoms (3). Hyperdopaminergic state of dopamine D2 receptor in the mesolimbic area is related to positive symptom prognosis, while hypodopaminergic dysregulation of the prefrontal cortex leads to negative symptoms (1). Only a decade ago, the focus from positive symptoms has shifted to the negative symptoms. Since then, very few pharmaceutical agents have been studied that successfully met the therapeutic target of negative symptoms (4). Typical (first generation) and atypical (second and third generations) antipsychotics are primarily used to modulate the dopaminergic function (5). The mechanism of action of first generation antipsychotics (FGAs) lack preference-based blocking of dopamine pathway, resulting in extrapyramidal symptoms (dyskinesia, akathisia and tremors), hyperprolactinemia-associated sexual dysfunction and aggravation of negative symptoms (6). The second generation antipsychotics (SGAs) are combined D2 and serotonin 5-HT_2A_ receptor antagonists with lower risk for developing extrapyramidal symptoms (EPS) (7). Second generation antipsychotics are effective for negative symptoms, but result in several treatment-emergent side effects including diabetes, ketoacidosis, weight gain, hyperlipidemia, hypercholesterolemia, hypertriglyceridemia, hypertension and metabolic syndrome (6, 8, 9).

Negative symptoms of schizophrenia have been consistently related to poor treatment outcome. A well-tolerated and long-term effective treatment option for negative symptoms is one of the most important unmet needs that is to be addressed. Globally approved third generation antipsychotic (TGA), cariprazine is distinct from other antipsychotics and has partial agonist activity at dopamine D3/D2 and serotonin 5-HT_1A_ receptors (10). Because of its dopamine-dependent partial agonism to D2 and D3 receptors, cariprazine is less likely to cause weight gain, metabolic disorder and hyperprolactinemia (6). This review attempts to outline the safety and efficacy of cariprazine for the treatment of predominant negative symptoms of schizophrenia.

## 2 Epidemiology of schizophrenia

According to World Health Organization (2022), schizophrenia affects about 24 million people worldwide, i.e., 1 in 300 (11). It is one of the top 15 causes of global disability (12). In India, the prevalence of schizophrenia is 1.5-2.5/1000 people, with an annual rate of 0.35-0.38/1000 and 0.44/1000 people from urban and rural area, respectively (13). Many factors including migration, drug abuse, urbanicity (stress, noise and pollution), childhood traumas, psychosocial factors, infections, cannabis use, birth during winter (high chances of respiratory infections and inadequate vitamin D synthesis), and obstetric complications during fetal, childhood, adolescence and early adult life can increase the risk of developing schizophrenia (14). The onset of schizophrenia mostly occurs during late adolescence and early adulthood, and happens earlier in males (average age 18 years) than females (average age 25 years) (1). Late onset of schizophrenia (after the age of 44 years) accounts for 15-20% of all cases (15).

## 3 Overview of negative symptoms of schizophrenia

Prevalence of negative symptoms is 75% in patients having schizophrenia and 68% in patients with schizoaffective disorder (16). Sometimes negative symptoms appear before the onset of positive symptoms (73% prevalence) or in the same month of developing positive symptoms (20% prevalence) (17). Ninety percent patients with first psychotic episode can develop at least one negative symptom, whereas 35-70% can still suffer from negative symptoms post-treatment (17).

**Figure 1:**
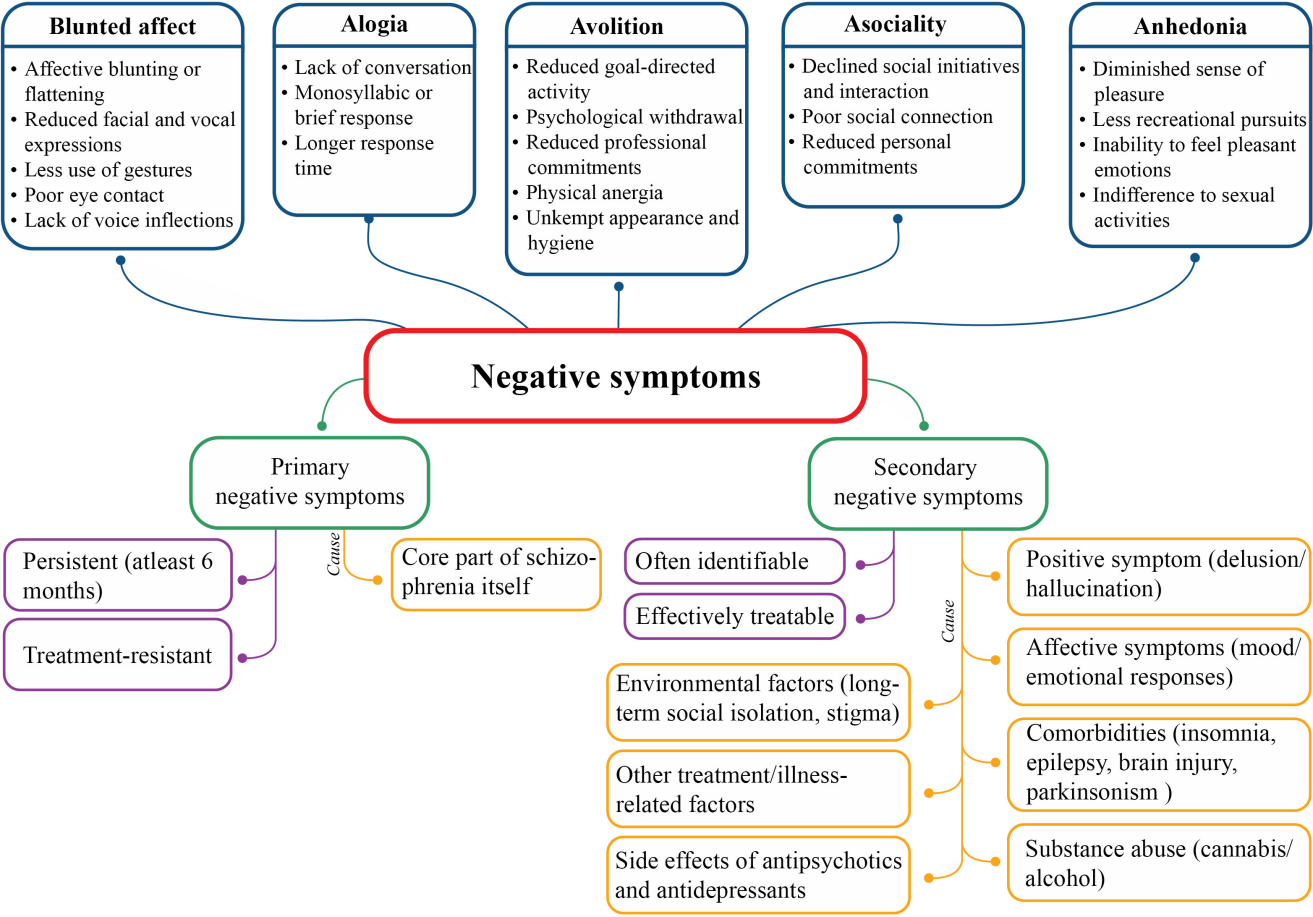
Nature, classification and etiology of negative symptoms.

The five constructs of negative symptoms are blunted affect, alogia, anhedonia, asociality and avolition (3) that cluster into two domains: the expressive domain (blunted affect and alogia) and the experiential domain (anhedonia, asociality and avolition); latter has a larger effect on the real-world functioning (18). Moreover, negative symptoms can be primary or secondary depending on their etiology (**Figure 1**). Primary negative symptoms for more than one year manifest deficit syndrome of schizophrenia and patients without these symptoms are considered to suffer from non-deficit schizophrenia (19). The severity of negative symptoms is also described by persistent (persisting over time, in spite of antipsychotic treatment), predominant (greater severity than co-occurring positive symptoms) and prominent (at least three moderate symptoms or two severe symptoms) negative symptoms (17, 20).

Pathology of negative symptoms includes decreased dopamine transmission in mesocortical pathways, along with decreased serotonergic, glutamatergic and noradrenergic transmission. Of note, hypodopaminergic functioning in the prefrontal lobe and additional mesolimbic structures are responsible for diminished motivation and reward-related processes, leading to negative symptoms (17, 21). Inhibition of glutamate neurotransmission after antagonizing N-methyl-D-aspartate (NMDA) receptors may results in negative symptoms of schizophrenia (17). Brain regions associated with expressive domain are rostral anterior cingulate cortex, amygdala, and ventrolateral prefrontal cortex; and with experiential domain are dorsal and ventral striatum, dorsolateral prefrontal cortex, anterior cingulate cortex and orbitofrontal cortex (21, 22).

Genetic factors, prenatal complications and poor premorbid adjustments prior to development of psychotic illnesses are contributing factors for onset of negative symptoms (17). Males are more prone to develop negative symptoms, especially anhedonia and avolition (23). Negative symptoms greatly affect disease prognosis, physical and psychological health, and personal and social relationships (24–28), as reduced functioning of mental health, health utility and expert-rated quality of life were reported (29). During early phase of the syndrome, negative symptoms increase the risk of self-harm that can persist up to 7 years since first psychiatric visit, and impact different domains of real-life functioning (18). Level of negative symptoms in elderly population is equivalent to that of younger schizophrenia population (30). Anxiety and depression are the central symptoms of population with predominant negative symptoms, hence clinicians must pay attention to these symptoms too, and not only to negative symptoms (31).

## 4 Assessment of negative symptoms of schizophrenia

Quantitative (frequency, duration and intensity) and qualitative (difference between anticipatory and consummatory aspects of anhedonia; or difference between behavioral and experiential aspects) aspects of negative symptoms is assessed using validated instruments (18). Different scales for standardized assessments of negative symptoms which are used either by professionals or by the patient (17, 20) is presented in **Table 1**.

**Table 1:** Various assessment scales for negative symptoms of schizophrenia.

Scales	Assessment	Reliability/Validity	Limitations
Brief Psychiatric Rating Scale (BPRS)	Includes 18 or 24 items with 0-6 rating	Assess all symptoms of schizophrenia	•Low inter-rater agreement •Internal inconsistency
Positive and Negative Syndrome Scale (PANSS)	Includes 30 items (7 positive, 7 negative and 16 general psychopathology items) with 1 (no symptom) to 7 (severe symptom) rating	•Psychometric validity •Inter-rater reliability	•Only measures negative symptoms at a single time point •Poor evaluation of avolition, apathy and anhedonia •Includes symptoms like difficulty in abstract thinking or inattentiveness; nowadays the symptoms are considered irrelevant to the negative symptoms •Difficulty in differentiating secondary negative symptoms
PANSS negative subscale	Includes 7 items: oN1: Blunted affect oN2: Emotional withdrawal oN3: Poor rapport oN4: Passive/apathetic social withdrawal oN5: Difficulty in abstract thinking oN6: Lack of spontaneity and flow of conversation oN7: Stereotyped thinking	•Internal consistency •Test-retest reliability •Inter-rater reliability	•Poor evaluation of avolition, apathy and anhedonia •Only behavioral observation is assessed
Scale for the Assessment of Negative Symptoms (SANS)	Includes 25 items that grouped into: oAlogia oEmotional blunting oAvolition-apathy oAnhedonia-asociality oDeficit of attention	Available in several languages	•Do not assess the five negative domains individually •Includes symptoms like difficulty in abstract thinking or inattentiveness; nowadays the symptoms are considered irrelevant to the negative symptoms •Difficulty in differentiating secondary negative symptoms
16-item Negative Symptom Assessment (NSA-16)	Includes 5 factors: oCommunication oEmotion/affect oSocial involvement oMotivation oRetardation	•Good psychometric properties •Excellent sensitivity •Separates patients with negative symptoms from without negative symptoms	•Only measures negative symptoms at a single time point •Includes symptoms like difficulty in abstract thinking or inattentiveness; •Includes behavior assessment that substantially overlaps with the functioning
4-item Negative Symptom Assessment (NSA-4)	Includes 4 items: oReduced speech oReduced emotion oReduced social drive oReduced interests Global rating of the overall impression of negative symptom severity is compared with a healthy person to provide anchor points for assessing severity of each symptom	•Good psychometric properties •Highly scalable and usable •Regardless of geographical region and the staffs operating the assessment, a uniform scaling is possible	–
Schedule for Deficit Syndrome (SDS)	Six negative symptoms with 0 (normal) to 4 (severely impaired) rating: oRestricted affect oDiminished emotional range oPoverty of speech oCurbing of interests oDiminished sense of purpose oDiminished social drive	•Inter-rater reliability •Good convergent validity •Greatest stability than other scales •Differentiates patients with deficit and non-deficit subtypes	Difficult to use in clinical practice
Clinical Assessment Interview for Negative Symptoms (CAINS)	Includes 13 items that measures all five domains of negative symptoms	•Good psychometric properties •Available in several languages	•Measurement is irrespective of primary or secondary negative symptoms or another aspect of the illness •Unavailability of trained raters
Brief Negative Symptom Scale (BNSS)	Includes 13 items that measures all five domains of negative symptoms	•Good psychometric properties •Available in several languages •Easy to use in clinical trials or clinical routines •Substantial advantages in identifying the domains in patients with predominant negative symptoms	•Measurement is irrespective of primary or secondary negative symptoms or another aspect of the illness •Unavailability of trained raters
Self-evaluation of Negative Symptoms (SNS)	Includes 20 items with 0 (strongly disagree), 1 (slightly agree) and 2 (completely agree) rating	•Self-assessment scale •Concise and easy to understand •Translated in several languages	–
Motivation and Pleasure Scale-Self-Report (MAP-SR)	Includes 15 items with 0-4 rating	•Self-assessment scale •Good psychometric properties	•Only assess motivation/pleasure dimension •Difficult for patients with memory impairment as the scale contains many questions

Brief Psychiatric Rating Scale (BPRS), Positive and Negative Syndrome Scale (PANSS) and Scale for the Assessment of Negative Symptoms (SANS) are used for diagnosis of deficit schizophrenia (20). In first episode of schizophrenia, patient’s phenomenological variety of negative symptoms can be evaluated with PANSS based experiential factor (‘poor rapport’, ‘passive/apathetic social withdrawal’, ‘active social avoidance’ and ‘lack of spontaneity’) and expressive factor (‘blunted affect’, ‘emotional withdrawal’ and ‘motor retardation’) (32). Brief Negative Symptom Rating Scale (BNSS) and Clinical Assessment Interview for Negative Symptoms (CAINS) have been developed by National Institute of Mental Health (NIMH) as the ‘next generation’ scale. In the USA, 16-item Negative Symptom Assessment (NSA-16) and SANS are recommended but not PANSS negative symptoms subscale, because of its inadequate coverage; whereas, the European drug authority has endorsed the use of SANS and PANSS negative symptoms subscale in clinical studies (9). Many European countries have approved the use of Self-evaluation of Negative Symptoms (SNS) scale (18).

## 5 Current treatment options for negative symptoms of schizophrenia and their limitations

Treatment of negative symptoms is challenging as no particular guidelines are available related to treatment algorithms and maintenance of the treatment; patients with treatment-resistant schizophrenia usually develop prominent negative symptoms (33, 34). The European regulatory guidelines and commentary issued by the US regulators have different perspectives with respect to treatment of schizophrenia, as the former recommend splitting negative symptoms from other domain of the disease, while the latter suggested lumping all the aspects of the disease together (9). The World Federation of Societies of Biological Psychiatry guidelines recommended use of FGA for secondary but not for primary negative symptoms of schizophrenia (35). Antipsychotic treatment is recommended by the American Psychiatric Association (APA) and the British Association for Psychopharmacology (BAP) for improvement and remission of both positive and negative symptoms; National Institute for Health and Care Excellence (NICE) and Canadian Psychiatric Association (CPA) suggested this treatment approach for improving functioning and quality of life. Reduced hospitalization and mortality with antipsychotic therapy are demonstrated by APA and CPA (34). United Nations High Commissioner for Refugees (UNHCR) recommended switching from FGA to SGA in case of ineffective treatment of negative symptoms (34). Likewise, the European Psychiatric Association guidelines recommended switching to SGA in patients not responding to FGA, along with social skill training and psychosocial rehabilitation (20).

First generation antipsychotics exhibits narrow efficacy spectrum in managing negative symptoms of schizophrenia (7). Second generation antipsychotics were introduced in the late 80s (24) with a promise to yield higher treatment efficacy, better receptor binding properties and lower side effects compared to FGAs (36). Significant difference in the pharmacological properties and side effect profiles exist between FGAs [fluphenazine, haloperidol, perphenazine and pimozide (D_2_ antagonists) and chlorpromazine, loxapine, thioridazine and trifluoperazine (D_2_ and 5-HT_2_ antagonists)], SGAs [iloperidone, lurasidone, olanzapine and ziprasidone (D_2_ and 5-HT_2_ antagonists), asenapine, clozapine, paliperidone and risperidone (5-HT_2_, D_2_ and norepinephrine α_2_ antagonist) and quetiapine (D_2_ and 5-HT_2_ antagonist and norepinephrine transporter reuptake inhibitor)], and TGAs [aripiprazole and brexpiprazole (D_2_ and 5-HT_1A_ partial agonist and 5-HT_2A_ antagonists)] (37, 38). Clozapine is considered as the best evidence-based therapeutic option for treatment-resistant schizophrenia (39). Higher efficacy of clozapine than other SGAs is reported for management of schizophrenia and schizophrenia-like psychoses (40). Despite its efficacy, 40% patients with treatment-resistant schizophrenia were reported to be non-respondent to clozapine treatment (41). Nielsen et al. reported improvement in negative symptoms after treatment with aripiprazole due to its partial D2 receptor agonist effect; however, no improvement in cognitive functions was found (42). Another study on patients with schizophrenia-spectrum disorders reported lower efficacy of aripiprazole in terms of improvement in PANSS negative score and CGI-S score (43). Although brexpiprazole has shown greater efficacy in improving negative symptoms (44), but common adverse effects associated with brexpiprazole are akathisia, headache, somnolence, weight gain and altered triglyceride level. Long-term risk and benefits of brexpiprazole are also not well-established (45).

Treatment-emergent adverse events are frequent with SGAs that commonly include akathisia, EPS, weight gain, sedation, insomnia, hyperprolactinemia and metabolic changes (46). Other adverse events include periorbital edema, parotitis, (inflammation of parotid gland/s) and pseudopheochromocytoma, i.e., severe paroxysmal hypertension (39). Lobos et al. reported higher incidence of akathisia with olanzapine, elevated glucose, triglycerides and prolactin levels with olanzapine and clozapine, hypercholesterolemia and hypersalivation with clozapine and low sexual drive with clozapine and risperidone treatment (40). Clozapine is also associated with other side effects viz. EPS, agranulocytosis, drooling, sedation, headache, dizziness, tremor, tachycardia, lengthening of corrected QT (QT_C_), weight gain, hypotension, visual abnormality, sweating, dry mouth, constipation, dyslipidemia and flexural intertrigo (39). Increase in prolactin level and EPS with amisulpride treatment and weight gain and elevated serum lipid and prolactin levels with amisulpride, aripiprazole, and olanzapine treatment were reported (43, 47). Additionally, evidence-based international guidelines revealed that SGAs have only moderate effect on negative symptoms; antidepressants and glutamatergic compounds are necessary to use additionally to overcome the disease burden (9). Schizophrenia patient data from 20 placebo-controlled trials reported prominent negative symptoms (8-33.1%), predominant negative symptoms (14.9%) and European Medicines Association (EMA) criteria-based negative symptoms (12.2-45.5%) even after 6 weeks of active treatment with SGA (48).

Poor outcomes with FGAs, and major side effects and inadequate response to SGAs leave a gap regarding the most appropriate treatment of negative symptoms, which is a long-standing challenge for schizophrenia management. Recently, a review on mental health care in central and eastern Europe suggested that many countries across the Europe have incorporated cariprazine as the first-line treatment for negative symptoms (49). Both the EMA and the US Food and Drug Administration (FDA) have approved cariprazine for schizophrenia management (50). The position statement of Polish Psychiatric Association on the use of D2/D3 receptor partial agonists highlighted the benefits of cariprazine in the management of predominant and persistent negative symptoms (51).

## 6 Cariprazine: a novel third generation antipsychotic

Cariprazine was approved in 2015 by the US FDA and later in 2018 in the UK (5, 7). The antipsychotic is approved in the US for treatment of schizophrenia, mania or mixed episodes, and depressive episodes related to bipolar I disorder and as adjunctive therapy to anti-depressants for the treatment of major depressive disorders, whereas in Europe it is approved for treatment of schizophrenia (26). Midlands and Lancashire Commissioning Support Unit 2019 has also recommended cariprazine for the treatment of predominant negative symptoms of schizophrenia (52). In 2021, India’s national regulatory body for cosmetics, pharmaceuticals and medical devices, Central Drugs Standard Control Organization (CDSCO) approved cariprazine for the treatment of schizophrenia, manic or mixed episodes associated with bipolar I disorder (53).

Cariprazine is available in capsule form with doses of 1.5, 3, 4.5, or 6 mg for schizophrenia treatment (6). At clinically relevant doses, cariprazine appeared to have higher occupancies of D2 and D3 receptors (54). Cariprazine dose of 1.5 mg/day results in 69% occupancy of both D2 and D3 receptors, and 3 mg/day for 14 days leads to 90% occupancy, suggesting adequate efficacy (6). Efficacy, tolerability and safety of cariprazine in patients having acute exacerbation of schizophrenia is established at a daily dose of 3 or 6 mg (55). More rapid onset of action (by 1 to 2 weeks) is achieved at a daily cariprazine dose of ≥3 mg than 1.5 mg; however, efficacy of cariprazine at 6^th^ week remains same with both higher and lower doses (56). Improvement in PANSS total score and CGI-S with cariprazine was reported at a dose of 1.5, 3 and 4.5 mg/day in one study (57), and at 3-6 or 6-9 mg/day in another study (58). Cariprazine dose of 4.5–6 mg/day improves negative symptoms (26); 3-6 or 6-9 mg/day improves PANSS and CAINS negative symptom scores (54) and 3, 6 or 9 mg/day lowers the chances of relapse (59). Cariprazine is also effective for patients with schizophrenia and concomitant substance use disorder, as it appeared to reduce cravings of illicit drugs/alcohol in such patients (60).

### 6.1 Unique aspect of cariprazine’s pharmacology

Cariprazine is a potent D2/D3 partial agonist with preferential binding to D3 receptors (61). This differs from two other TGAs like aripiprazole and brexpiprazole, by its distinct receptor-binding characteristics not only at dopamine D2/D3 receptors, but also at serotonin 5HT1A, 5HT2B, 5HT2A, 5HT2C, and histamine H1 receptors (62). Cariprazine acts as an antagonist when dopamine activity is normal and as partial agonist when the activity is low, depending on the available dopamine (63). This feature of cariprazine is proven effective for treatment of predominant primary negative symptoms of schizophrenia (24, 64). It is especially recommended for elder patients, as cariprazine results in procognitive and antidepressant effects due to its partial agonism towards D2/D3 receptors (7). Cariprazine also acts as an antagonist to 5-HT_2B_ and a partial agonist to 5-HT_1A_. Its strong affinity towards 5-HT_1B_ receptor is the reason for reduced EPS and akathisia; however, the clinical relevance of antagonism to serotonin 5-HT_2B_ receptors is unknown. Partial agonism of cariprazine to 5-HT_1A_ receptors lowers depressant effects of schizophrenia, and weak antagonism to 5-HT_2C_ and H1 receptors reduces risk of weight gain, metabolic abnormalities and sedation than olanzapine and quetiapine (63). Additionally, cariprazine has a lower or negligible affinity for noradrenergic, histaminergic, and cholinergic receptors (65). Because of its lower inhibition of dopaminergic neurotransmission in the striatum, cariprazine has lower risk of developing EPS than other atypical antipsychotics (63). The receptor binding affinities of different anti-psychotics in comparison with cariprazine is shown in **Table 2** (5, 36, 66–69).

**Table 2:** Receptor binding affinities of cariprazine in comparison to other antipsychotics.

Receptors → Antipsychotics ↓	Binding affinities (nM Ki)						
	D1	D2	D3	5-HT_1A_	5-HT_2A_	5-HT_2C_	5-H_T7_
SGAs							
*Asenapine*	–	1.7	1.8	2.51	0.071	0.035	0.12
*Clozapine*	192.5	190	280	120	5.4	9.4	–
*Iloperidone*	–	8.3	10.5	–	–	–	–
*Lurasidone*	–	0.66	15.7	6.8	2	–	0.495
*Olanzapine*	52.5	30.8	38.1	2720	4.9	14	104
*Paliperidone*	–	1.4	2.6	590	1	19	6.8
*Quetiapine*	741.3	437	394	320	200	1406.3	1800
*Risperidone*	267.0	4.9	14	420	0.48	33	3
*Ziprasidone*	–	4.75	7.3	112	0.73	4.1	–
TGAs							
*Cariprazine*	–	0.49^**^	0.085^**^	2.6^**^	18.8^*^	134^*^	111^*^
*Aripiprazole*	387	2.3	4.6	5.6	8.7	18.7	39
*Brexpiprazole*	–	0.3	1.1	0.12	0.47	34	3.7

Antagonist^*^; Partial agonist^**^; Ki < 1: Very strong association; Ki < 10: Strong association; Ki < 100 Moderate association; Ki < 1000: Weak association.

SGAs, Second generations antipsychotics; TGAs, Third generations antipsychotics.

Greater affinity for D3 receptor together with actions of serotonin receptors makes cariprazine a potential antipsychotic for alleviating the negative symptoms. Moreover, these symptoms are responsible for poor social functioning, impacting patient’s daily functioning and quality of life. Efficacy of cariprazine is well-established in treating negative as well as cognitive and affective symptoms of schizophrenia, thus improving social behavior of the patient. For this reason cariprazine is regarded as a ‘socializing drug’ (70). In addition to ten-fold higher affinity for D3 receptors, cariprazine adds exceptional values to schizophrenia management because of its long half-life and broad-spectrum efficacy and safety (3, 5, 71). A remarkably longer half-life of the active metabolites of cariprazine, desmethyl cariprazine (DCAR) and didesmethyl cariprazine (DDCAR), of 2-4 days and 1-3 weeks (67) respectively, prevents patients from experiencing incidence of relapse even after accidentally missing dose. Early and late efficacy are offered by DCAR and DDCAR, respectively; with both depicting mean concentrations of 400% and 30% respectively even after 12 weeks of cariprazine administration (56). Cariprazine provides a significantly longer time to relapse (defined by occurrence of psychiatric hospitalization, worsening of symptom scores, aggression or violence or suicidal tendency) and lower chance of relapse (59).

It may be noted that, because cariprazine and its active metabolites have long half-lives, the active moiety would take several weeks to reach steady state; this is unlikely to be a problem as efficacy has anyway been demonstrated in clinical trials. However, because of the long half-lives, the active moiety would take long to wash out. This could be positive if patients do not take the drug for one or more days or temporarily discontinue the treatment, as the drug is still in the body. The long half-lives also obviate the risk of a drug discontinuation syndrome. However, the long half-lives could be negative if rapid reduction of blood levels is desired, as when patients experience adverse effects or become pregnant (72).

### 6.2 Clinical evidences on cariprazine in management of negative symptoms of schizophrenia

#### 6.2.1 Efficacy of cariprazine treatment

The broad-spectrum efficacy of cariprazine in treatment of schizophrenia and predominant negative symptoms in terms of reduction in blunted affect, emotional withdrawal, passive/apathetic social withdrawal, poor rapport and difficulty in abstract thinking according to PANSS score is established (3). Although antipsychotic monotherapy is recommended for schizophrenia treatment, with the evidence of efficacy of polypharmacy in the real world, monotherapy is often challenged (6). On the other hand, adverse effects of using multiple antipsychotics disapproved the idea of polypharmacy (20). Available findings on cariprazine monotherapy or adjunctive therapy for negative symptom treatment are summarized in **Table 3**. Németh et al. conducted a phase III randomized trial in eleven European countries, and found a significant improvement in predominant negative symptoms with cariprazine than risperidone, starting from ~3 months of treatment, as well as a greater treatment adherence. Moreover, the improvement was independent of EPS, positive and depressive symptoms (71). A recently published study found that a single trajectory best described improvement of negative symptoms with cariprazine: there was steady improvement all through the trial with most improvement occurring during the first 4 weeks (76). Another study demonstrated effectiveness of cariprazine monotherapy in reducing PANSS negative subscale items and PANSS-derived factors by week 26; in comparison to risperidone, the efficacy of cariprazine in negative symptom improvement was an exclusive effect of the antipsychotic only (3). Cariprazine showed to have higher improvement in moderate/severe negative symptoms in patients with acute schizophrenia compared to aripiprazole (24). A lower number needed to treat (NNT) indicates therapeutic effects of a drug compared to the comparator, based on the visible improvements (77). The NNT of cariprazine is lower than risperidone (n=3 vs. 6) and aripiprazole (n=3 vs. 19) in achieving PANSS factor score for negative symptoms, suggesting that cariprazine dose of 1.5–3 mg/day is sufficient to accomplish positive outcomes than risperidone and aripiprazole (26). A small uncontrolled, open label study in patients with early psychosis found that the mean negative PANSS score decreased from 26 (at baseline) to 11 (at 6 months) in patients who tolerated cariprazine (1.5-3.0 mg/day) and responded to it (78). Treatment-resistant or drug-naïve schizophrenia has shown improvement with cariprazine treatment (1, 75). Steady state of paranoid delusions and aggressiveness was achieved with 2 weeks of cariprazine treatment (79). Cariprazine as adjunctive or monotherapy also resulted in remission of negative symptoms (5, 25, 64, 74).

**Table 3:** Summary of clinical evidence of cariprazine for management of negative symptoms of schizophrenia.

Reference	Study type	Sample demographics	Diagnosis/Symptoms	Dose of antipsychotics	Monotherapy/Adjunctive therapy	Outcomes of cariprazine treatment	Side effects of cariprazine
(71)	Randomized controlled trial	Cariprazine group, n=230 Risperidone group, n=230 Age: 18–65 years	Schizophrenia, predominant negative symptoms and low levels of positive symptoms	•Cariprazine 3, 4.5 or 6 mg/day for 26 weeks •Risperidone 3, 4 or 6 mg/day for 26 weeks	Monotherapy	•Improved CGI-I and CGI-S scales •Improved PANSS factor score for negative symptoms •Improved predominant negative symptoms	Insomnia, akathisia, worsening of schizophrenia, headache, anxiety
(3)	Post-hoc analysis	n=456 Age: 18-65 years	Schizophrenia, negative symptoms	•Cariprazine 3, 4.5, or 6 mg/day •Risperidone 3, 4.5, or 6 mg/day	Monotherapy	•Improved PANSS negative subscale with cariprazine	None
(26)	Post-hoc analysis	Age: 18–60 years	Schizophrenia, moderate/severe negative symptoms	•Cariprazine 1.5, 3, 4.5 or 6 mg/day •Risperidone 4 mg/day •Aripiprazole 10 mg/day	Monotherapy	•Greater improvement in moderate/severe negative symptoms with cariprazine	Discontinuations due to adverse events with cariprazine high-dose
(54)	Open-label non-controlled study	n=60 Age: 35.6± 9.1 years	Schizophrenia, predominant negative symptoms	Cariprazine, initial dose: 1.5 mg, (weekly upward titration by 1.5 mg up to 6 mg) for 28 days	Monotherapy	•Improvement in 75% patients •Improved PANSS-negative scale and CAINS score •No change in depression	Akathisia, persistent insomnia, and anxiety
(73)	Open-label observational study	n=116 Age: 37.4 ± 11.3 years	Schizophrenia, negative symptoms	Cariprazine1.5 mg/day or 3 mg/day or 4.5 mg/day or 6 mg/day	Monotherapy	•Overall improvement in disease severity •Significantly improved negative symptoms •Improved CGI-I and CGI-S scores in over 70% of patients •>70% doctor satisfaction regarding drug effectiveness and tolerability	Akathisia, anxiety, parkinsonism, dizziness, lethargy, insomnia and sleep disorder
(5)	Case series	**Case 1:** 34 year old female **Case 2:** 60 year old female **Case 3:** 23 year old male **Case 4:** 51 year old male **Case 5:** 28 year old male	**Case 1, 2, 3 and 5:** Paranoid schizophrenia **Case 4:** Schizoaffective disorder	**Case 1:** Clozapine 275 mg/day + cariprazine 1.5 mg/day **Case 2:** Clozapine 250 mg/day + cariprazine 1.5 mg/day **Case 3:** Clozapine up to 325 mg + cariprazine 1.5 mg/day **Case 4:** Clozapine 600 mg/day + cariprazine 1.5 mg/day **Case 5:** Clozapine 700 mg/day + cariprazine 1.5 mg on alternative days	Adjunctive therapy (Cariprazine+clozapine)	Reduced negative symptoms	None
(74)	Case series	**Case 1:** 29 year old male **Case 2:** 21 year old male **Case 3:** 32 year old male	Schizophrenia, persistent negative and cognitive symptoms	**Case 1:** Cariprazine 1.5 mg/day for 3 days, increased up to 3 mg/day + clozapine up to 800 mg/day for 6 months **Case 2:** Cariprazine 1.5 mg/day for 1 week, increased to 4.5 mg/day for 8 months **Case 3:** Cariprazine 4.5-6 mg/day for 9 months	**Case 1:** Adjunctive therapy (Cariprazine + clozapine) **Case 2 and 3:** Monotherapy	**Case 1:** Improved psychomotor drive and mood **Case 2:** No recurrence of positive symptoms **Case 3:** Improved negative symptoms and no psychotic symptoms	None
(75)	Case report	25 year old male	Treatment-resistant schizophrenia: Debilitating psychotic symptoms, failed to respond to risperidone, aripiprazole or clozapine	Cariprazine 1.5 mg/day for 1 week, increased to 3 mg/day	Monotherapy	•Improved positive and negative symptoms •Improved social functioning	None
(64)	Case report	45 year old male	Long-standing treatment-resistant schizoaffective disorder	Cariprazine 1.5 mg/day for 4 days, 3 mg/day for next 12 days, then 4.5 mg/day	Adjunctive therapy (Clozapine + cariprazine)	•Near-complete remission of persistent negative symptoms •Improved quality of life	None
(25)	Case report	**Patient 01:** 37.5 year old male **Patient 02:** 33.5 year old male **Patient 03:** 36 year old female	Schizophrenia, persistent negative symptoms	**Patient 01:** Risperidone was gradually tapered off and cariprazine was initiated at 1.5 mg/day, titrated to 6 mg/day (for 15 days) **Patient 02:** Olanzapine 10 mg/day was gradually tapered off to initiate cariprazine titrated up to 6 mg/day (for 20 days) **Patient 03:** Quetiapine 600 mg/day was switched to cariprazine 1.5 mg/day titrated to 6 mg/day (for 22 days)	Monotherapy	•Improvement in negative symptoms, global functioning, and CGI after 12 weeks of cariprazine treatment •Antipsychotic switch from various antipsychotics to cariprazine was well tolerated in all cases	None
(1)	Case report	23 year old female	Schizophrenia: Psychosis and severe negative symptoms	Cariprazine, initial dose: 1.5 mg/day, titrated to 4.5 mg/day over a 2 week period and then 3 mg/day for 52 weeks	Monotherapy	Improved PANSS and CGI scores and psychological tests; effect lasting for >12 months	Mild EPS after 8 weeks

CAINS, Clinical Assessment Interview for Negative Symptoms; CGI-I, Clinical Global Impressions-Improvement; CGI-S, Clinical Global Impressions-Severity; EPS, Extrapyramidal Symptoms; PANSS, Positive and Negative Syndrome Scale.

#### 6.2.2 Safety of cariprazine treatment

The most common adverse reactions with cariprazine treatment (incidence rate of ≥ 5%) are EPS and akathisia in patients with schizophrenia; EPS, akathisia, dyspepsia, vomiting, somnolence, and restlessness in bipolar mania; nausea, akathisia, restlessness and EPS in bipolar depression; and akathisia, restlessness, fatigue, constipation, nausea, insomnia, increased appetite, dizziness, and EPS in adjunctive treatment of major depressive disorder (80). Previous studies on safety and tolerability of cariprazine monotherapy demonstrated that treatment with cariprazine is generally well-tolerated and lowers total cholesterol, low-density lipoprotein, high-density lipoprotein and triglyceride levels in patients with schizophrenia (81, 82). Long term safety of cariprazine monotherapy in adults with schizophrenia is established by Cutler et al.; safety and tolerability remained consistent up to one year (83). Normal electrocardiogram (ECG) and occurrence of mild/moderate treatment-emergent adverse events (akathisia, insomnia, headache and weight increased; anxiety and tremor) over the course of 53 weeks of cariprazine treatment was found in patients with acute exacerbation of schizophrenia. Safety and tolerability of cariprazine in terms of vital signs, body weight, clinical laboratory tests and ECG has been recorded by a *post hoc* analysis including four short (6 weeks) and four long (≥6 months) term studies. The study reported that cariprazine has a good safety profile and is well-tolerated with lower rates of treatment-emergent adverse events, independent of the treatment durations (84). Another *post hoc* analysis of pooled data from three short term (6 weeks) trials recorded safety of cariprazine in both early (<5 years) and late (>15 years) stage schizophrenia patients. Although insomnia, akathisia, EPS and headache occurred in both groups but discontinuation from the study was not related to the adverse events (85). Insomnia, akathisia, constipation, anxiety, nausea and vomiting are reported to occur with cariprazine treatment in patients with negative symptoms of schizophrenia (**Table 3**). However, the side effects have lower occurrence rate than other available SGAs (86). Discontinuation of cariprazine due to treatment-emergent adverse events is as low as 9% (62). Despite the recorded side effects, ~70% clinicians rated cariprazine’s effectiveness and tolerability as ‘satisfactory’ or ‘very satisfactory’ (73). If long-term efficacy and tolerability is the chief concern with negative symptom treatment then cariprazine may be used as the first-line treatment for both prominent negative symptoms and severe positive symptoms (25). Observing the clinical changes in negative symptoms with cariprazine, it can be suggested as a good treatment option for predominant negative symptoms of schizophrenia.

## 7 Summary

**Figure 2:**
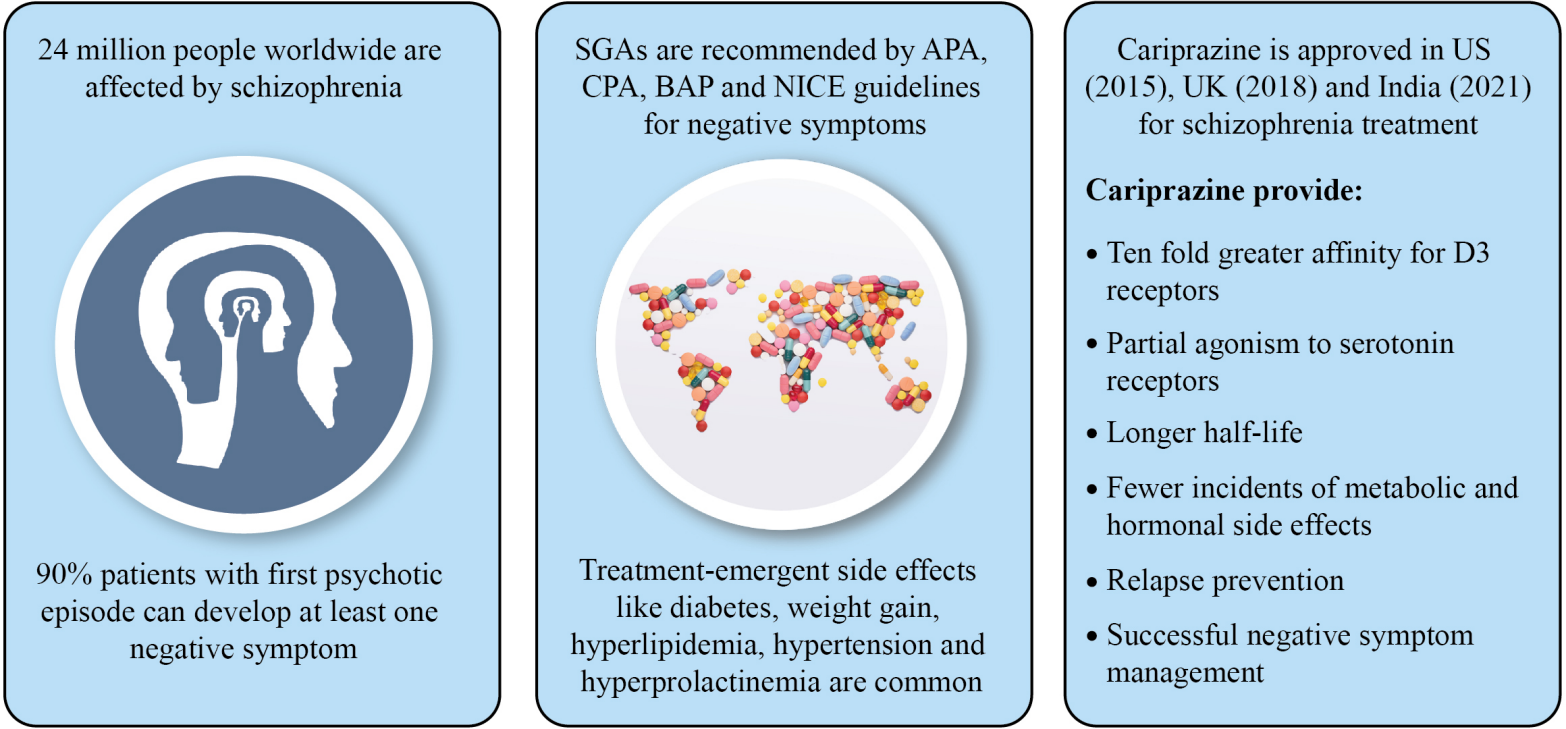
Summary of the study.

This review article provides new insights on the possible use of cariprazine for negative symptom management (**Figure 2**). In a nutshell, negative symptoms of schizophrenia hinder patient’s quality of life and treatment options are limited. Antipsychotic management of negative symptoms is recommended by various international guidelines. However, FGAs are ineffective for treatment of negative symptoms when they are secondary to positive symptoms, and SGAs have partial benefits on negative symptoms due to frequent incidence of treatment-related side effects. Cariprazine, a recently approved antipsychotic, has high affinity and occupancy for D2/D3 receptors, partial agonism to 5-HT_1A_ and antagonism to 5-HT_2B_ receptors, and longer half-life which is efficacious in management of patients with negative symptoms of schizophrenia. The drug appears to be superior to available SGAs with lower incidence of metabolic disorders and relapse. Therefore, cariprazine can be used as a viable alternative to other antipsychotics for predominant negative symptom treatment. More clinical trials need to be conducted to confirm the beneficial effect of cariprazine for treatment of negative symptoms over other antipsychotics.

## Author contributions

PS: Validation, Writing – review & editing. PD: Data curation, Investigation, Writing – original draft. AS: Data curation, Investigation, Writing – original draft. SD: Data curation, Investigation, Writing – original draft. CK: Data curation, Investigation, Writing – original draft. AM: Validation, Writing – review & editing. SM: Validation, Writing – review & editing. CA: Supervision, Validation, Writing – review & editing.
